# Taxing Physicians

**Published:** 1883-01-20

**Authors:** 


### *TAXING PHYSICIANS.*

The specific tax of $10 in the state of Georgia upon the medical practitioner has always seemed to us to be unjust. Not that the medical man should claim exemption from bearing his share of the burdens of government, but that reasons exist why he should be relieved from this tax upon his profession. He pays upon his property and upon his income as do other men, and it is claimed that something should be paid upon his profession, which may be said to be his capital, in order to cover those cases wherein the party has no property, and yet this is not done upon all other professions, as, for instance, the Civil Engineer, the school teacher, the architect, etc.

But there is a stronger reason why the physician should be exempted from this specific tax, and that is because of the great amount of gratuitous labor that he performs, the benefit of which acrues to the public. No other class of men do so much for the poor as the medical man. It is well known that the state does not now, and never did, make adequate provision for the indigent sick. At all times, often in the dark hours of night, when others are asleep, and in all sorts of weather, the physician passes from one scene of distress to another, injuring his health and risking life in behalf of the sick, who, in a great many instances, are unable to compensate him either for his labor or the medicines which he furnishes. To this he is impelled both by public sentiment and by the dictates of humanity. To say, as is often done, that he has voluntarily chosen a profession to which such duties are incident, furnishes no reason for that demand of public sentiment which exacts it of him, and is in itself a contemptible argument. Certainly there exists in it no proper ground for increasing his burdens by a specific tax. There is also another imposition to which the medical man is subject, which of itself furnishes a strong reason why he should be relieved of the specific tax, and that is, that he is compelled to give expert testimony before the courts without compensation.

Efforts have been vainly made in past years to secure the repeal of these unjust exactions. Yet we think the profession should not abandon their efforts in these matters, but that they should combine their influence against them throughout the state, and persevere until we are relieved. W.

